# Azole Resistance of *Aspergillus fumigatus* in Immunocompromised Patients with Invasive Aspergillosis

**DOI:** 10.3201/eid2201.150848

**Published:** 2016-01

**Authors:** Alexandre Alanio, Blandine Denis, Samia Hamane, Emmanuel Raffoux, Régis Peffault de Latour, Jean Menotti, Sandy Amorim, Sophie Touratier, Anne Bergeron, Stéphane Bretagne

**Affiliations:** Groupe Hospitalier Saint-Louis-Lariboisière-Fernand-Widal, Assistance Publique-Hôpitaux de Paris, Paris, France (A. Alanio, B. Denis, S. Hamane, E. Raffoux, R. Peffault de Latour, J. Menotti, S. Amorim, S. Touratier, A. Bergeron, S. Bretagne);; Université Paris Diderot, Sorbonne Paris Cité, Paris (A. Alanio, R. Peffault de Latour, J. Menotti, S. Amorim, A. Bergeron, S. Bretagne);; National Reference Center of Invasive Mycoses and Antifungals, Institut Pasteur, Paris (A. Alanio, S. Bretagne);; Le Centre National de la Recherche Scientifique URA 3012, Paris (A. Alanio, J. Menotti, S. Bretagne)

**Keywords:** *Aspergillus fumigatus*, azoles, invasive aspergillosis, antimicrobial resistance, fungi, France, immunocompromised

**To the Editor:** First-line antifungal therapy for invasive aspergillosis (IA) is voriconazole, which is challenged by the emergence of azole resistance (*1*). A recent article reported a 3.2% prevalence of *Aspergillus fumigatus* isolates that are resistant to azole from 3,788 isolates screened in Europe (*2*). Of the 1,911 patients from whom the isolates were collected, IA developed in 10 (3 proven, 1 probable, 6 possible). Prevalence of azole-resistant *A. fumigatus* disease among patient populations at risk of IA was unavailable.

As described (*3*), we screened every *A. fumigatus* isolate recovered from respiratory specimens from patients with probable or proven IA in our hospital in Paris, France, during January 2012–December 2014. Every isolate recovered from 2% malt extract agar plates or Sabouraud dextrose agar slants (Bio-Rad, Marnes-la-Coquette, France) was incubated at 30°C and tested as individual isolates or multiple ones from a single sample by using itraconazole, voriconazole, and posaconazole Etest strips (bioMérieux, Marcy l’Etoile, France). Resistance was assessed for MICs >2.0 µg/µL for voriconazole and itraconazole and >0.25 µg/µL for posaconazole by using European Committee on Antimicrobial Susceptibility Testing clinical breakpoints for fungi (http://www.eucast.org/fileadmin/src/media/PDFs/EUCAST_files/AFST/Antifungal_breakpoints_v_7.0.pdf).

Every 4 months, a local multidisciplinary medical team classified each IA case by using the 2008 criteria established by the European Organization for Research and Treatment of Cancer/Invasive Fungal Infections Cooperative Group and the National Institute of Allergy and Infectious Diseases Mycoses Study Group (*4*). For 148 patients (127 with hematologic malignancies and 21 with other conditions), the team recorded 152 episodes: 9 proven and 143 probable IA episodes. Possible IA was not analyzed because of a lack of microbiologic criteria. For 51 probable IA episodes, galactomannan positivity in blood or bronchoalveolar lavage fluid samples was the only microbiologic criterion used for classification. Cultures of respiratory samples (i.e., bronchoalveolar lavage fluid, tracheal aspirate, and sputum) or biopsies were positive for 99 episodes: 68 with *A. fumigatus* isolates and 31 with other *Aspergillus* spp. isolates. Among the 68 *A. fumigatus* isolates, 1 (1.5%) associated with probable IA was resistant to azoles (*5*). The isolate harbored the TR_34_/L98H mutation (*5*), leading to a rate of IA caused by azole-resistant *A. fumigatus* of 0.7% (1/152) for total episodes recorded and 1% (1/99) for culture-positive episodes only. Nineteen (36%) of 53 culture-negative patients and 35 (37%) of 95 culture-positive patients died.

Azole resistance of *A. fumigatus* warrants specific surveillance in hospitals treating immunocompromised patients. Prevalence of resistant isolates can differ by hospital location and underlying disease (e.g., immunodeficiency vs. chronic lung diseases). When focusing on patients with probable or proven IA, we did not observe an emergence of azole-resistant *A. fumigatus* isolates during 2006–2009 (*3*) and 2012–2014 in France. Consequently, our center does not question the use of voriconazole as first-line treatment or of posaconazole as prophylaxis.
